# A Retrospective Overview of Enterovirus Infection Diagnosis and Molecular Epidemiology in the Public Hospitals of Marseille, France (1985–2005)

**DOI:** 10.1371/journal.pone.0018022

**Published:** 2011-03-18

**Authors:** Charlene Y. Q. Tan, Laetitia Ninove, Jean Gaudart, Antoine Nougairede, Christine Zandotti, Laurence Thirion-Perrier, Remi N. Charrel, Xavier de Lamballerie

**Affiliations:** 1 UMR190 Unite des Virus Emergents, Universite de la Méditerranee - Institut de Recherche pour le Développement, Marseille, France; 2 Federation de Microbiologie, Assistance Publique-Hopitaux de Marseille, Marseille, France; 3 LERTIM EA3283, Biostatistic Research Unit, Universite de la Mediterranee, Marseille, France; Institute of Infectious Disease and Molecular Medicine, South Africa

## Abstract

Human enteroviruses (HEV) are frequent human pathogens and, associated in particular with large outbreaks of aseptic meningitis. Here, we have compiled a database of clinical HEV isolates from the Public Hospitals of Marseille, from 1985 to 2005. Amongst 654 isolates that could be characterized by complete sequencing of the VP1 gene, 98% belonged to species HEV-B; the most frequently isolated serotypes were Echovirus E30, E11, E7, E6 and E4. The high incidence of E30 and the recent emergence of E13 are consistent with reports worldwide and peak HEV isolation occurred mostly in the late spring and summer months. The proportion of echoviruses has decreased across the years, while that of coxsackieviruses has increased. Stool (the most frequent sample type) allowed detection of all identified serotypes. MRC5 (Human lung fibroblasts) cell line was the most conducive cell line for HEV isolation (84.9% of 10 most common serotype isolates, 96.3% in association with BGM (Buffalo green monkey kidney cells)). Previous seroneutralization-based serotype identification demonstrated 55.4% accuracy when compared with molecular VP1 analysis. Our analysis of a large number of clinical strains over 20 years reinforced the validity of VP1 serotyping and showed that comparative p-distance scores can be coupled with phylogenetic analysis to provide non-ambiguous serotype identification. Phylogenetic analysis in the VP1, 2C and 3D regions also provided evidence for recombination events amongst clinical isolates. In particular, it identified isolates with dissimilar VP1 but almost identical nonstructural regions.

## Introduction

Human enteroviruses (*HEV*, family *Picornaviridae*) are small non-enveloped viruses with a single-stranded RNA genome of positive polarity. The genome is approximately 7.4 kb long. Four structural proteins, VP1 to VP4, are assembled to form the virion capsid of icosahedral symmetry. The most conserved regions of the enteroviral genome are the 5′non-coding region (5′NCR) and the RNA-dependent RNA polymerase [1]–[2]. 64 distinct human serotypes were previously identified on the basis of their pathogenic potential and neutralization by specific antisera. They were then later reclassified into four species based on sequence identity of the region coding for the VP1 capsid protein. The four species are (i) *HEV-A*, (ii) *HEV-B*, (iii) *HEV-C* including *Poliovirus* (PV), and (iv) *HEV-*D [3]–[4].

Laboratory detection of enteroviruses is performed today by the ‘gold standard’ method of a pan-enterovirus real-time RT-PCR in the 5′NCR, which allows the detection of all enteroviruses but not the identification of their serotype [5]. Conventional serotyping consists of neutralization tests with Lim Benyesh-Melnick antiserum pools raised against prototype strains [6]. Modern day serotype identification is based on virus isolation in cell culture and the nucleotide sequence of the region coding for VP1 protein. VP1 sequences from prototype strains have been demonstrated to correlate best with neutralization-based serotype, as it is the site of major epitopes associated with serotype-specific neutralization [7]–[8]. More recently, molecular protocols aiming at identifying the HEV serotype directly from clinical samples have been proposed [9]–[12].

HEV cause a wide spectrum of illnesses ranging from mild (hand, foot and mouth disease, gastroenteritis, acute haemorrhagic conjunctivitis) to severe and potentially life-threatening (acute flaccid paralysis) [13]–[14]. Most enteroviral infections are asymptomatic or subclinical but their neurotropism can cause serious central nervous system complications such as aseptic meningitis and encephalitis. Enteroviruses are the major cause of aseptic meningitis in both pediatric and adult populations [15]–[16]. There is currently no antiviral treatment available for HEV infection [17].

There is worldwide circulation of enteroviruses, except for poliovirus which remains endemic in only four countries (Pakistan, India, Nigeria and Afghanistan) [18]. Seasonal aseptic meningitis outbreaks due to non-polio enteroviruses have been noted to peak in summer till early autumn in the Northern Hemisphere [19]. While the prevalent serotype varies from year to year, with co-circulation of several serotypes a common observation, recent epidemics have been attributed primarily to Echovirus 30 (E30) [20]–[24]. The molecular mechanism for enterovirus evolution couples mutation due to viral polymerase error and homologous recombination by template switching [25]. The evolutionary overview of enteroviruses appears to be considered as genome fragments in a global reservoir, subjected to independent evolutionary forces and recombination events [26]–[28].

Here, we have compiled a comprehensive database of HEV isolated at the Public Hospitals of Marseille (AP-HM), France, spanning 1985 to 2005 with VP1 nucleotide sequences of clinical HEV strains. We systematically analyzed it for epidemiological information as well as trends in laboratory diagnostic techniques.

## Results

### Frequency of HEV isolation

**Figure 1:**
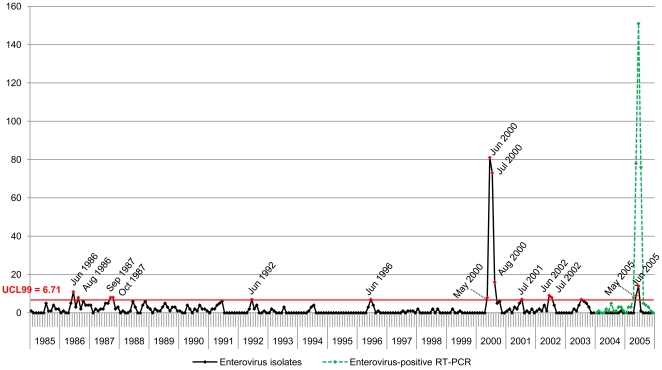
Monthly distribution of Enterovirus isolates (1985–2005), and Enterovirus-positive RT-PCR diagnostic cases (2004–2005). We identified 9 years of significantly high HEV isolation frequency during which the number of monthly isolates exceeded the upper control limit at 99% confidence level (UCL99 = 6.71, p<0.01). Peak isolation levels were mostly between May and August, except in 1987 in September and October. 2 epidemics were also recorded in 2000 and 2005.

Of 828 secondary cell cultures tested positive for HEV by immunofluorescence, 654 (79%) were successfully sequenced in the VP1 region and attributed their serotype. We identified 9 years with significantly high HEV isolation frequency during which the number of monthly isolates exceeded the upper control limit at 99% confidence level (UCL99 = 6.71, p<0.01) (**Figure 1**), 8 of which saw peak HEV isolation between May andAugust, in the late spring and summer months. The only exception was in 1987 when peak HEV isolation occurred in September and October, in the fall. Isolation levels in 2000 and 2005 were of great amplitude, and coincide with the occurrence of HEV epidemics in Marseille. HEV isolates in 2000 (n = 191) peaked in the summer months, with 93.2% (n = 178) occurring between May and August. 50.3% of the isolates in 2000 were attributed to the serotype E30 (n = 96), 15.7% to E13 (n = 30) and 7.9% to E11 (n = 15). In 2005, only 6.7% (n = 24) of all cases were isolated and typed as a result of a change in hospital diagnostic protocol. Nevertheless, the 2005 epidemic is evidenced by the number of cases diagnosed Enterovirus-positive with real time RT-PCR (n = 78, 151, 76 for May, June and July respectively). HEV isolates during the remaining peaks comprised numerous serotypes, including E30, E11, E7, E18 and CVB5, without clear predominance of any one serotype.

### Serotype patterns

**Figure 2:**
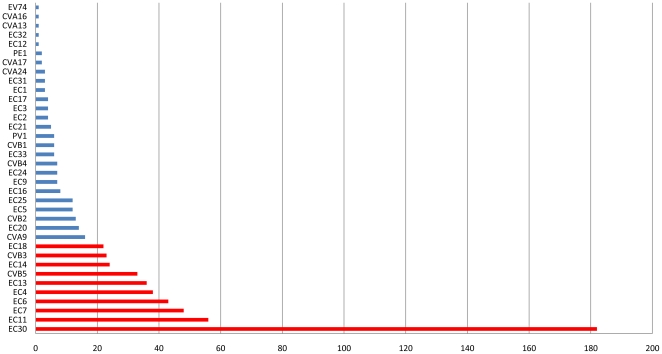
Distribution of Enterovirus serotypes isolated in Marseille, 1985–2005. The 10 most common serotypes isolated between 1985 and 2005 in Marseille account for 77.1% of all cases. Shown in red: (in decreasing order) E30, E11, E7, E6, E4, E13, CVB5, E14, CVB3 and E18. The top 5 most frequently encountered serotypes alone account for 56.7%.

Overall, the 10 HEV serotypes most commonly isolated between 1985 and 2005 all belong to the HEV-B species: in decreasing frequency, E30, E11, E7, E6, E4, E13, CVB5, E14, CVB3 and E18 (**Figure 2**) and account for 77.1% of isolates with known serotype. The 5 most frequently encountered serotypes, E30, E11, E7, E6 and E4 account for 56.7% of all isolates, and remain the most prevalent serotypes even after factoring out the 2000 and 2005 epidemics. HEV-B accounted for 98% of all isolates.

Long-term circulation patterns varied for individual serotypes. Some serotypes have disappeared from Marseille: the last reported cases of CVA13, CVA17, E1, E2, E3, E12, E14, E31, E32, EV74 and PV all precede 1992. On the other hand, other serotypes have appeared with varying frequencies: CVA24 has been isolated with extremely low frequency (4 isolates since 1996), in contrast with E13 which reappeared in 2000 as an epidemic serotype. Only a single E13 infection was reported in Marseille prior to the 2000 outbreak. Serotypes such as E30, E7, E6 and E4 demonstrate more endemic patterns, with persistent isolation levels over 20 years. E30 in particular, has a strong propensity for epidemic eruptions and was the most commonly identified enterovirus during seven years of the study period (1987, 1988, 1996, 2000, 2001, 2002 and 2005).

### Classification and Regression Tree (CART)

**Figure 3:**
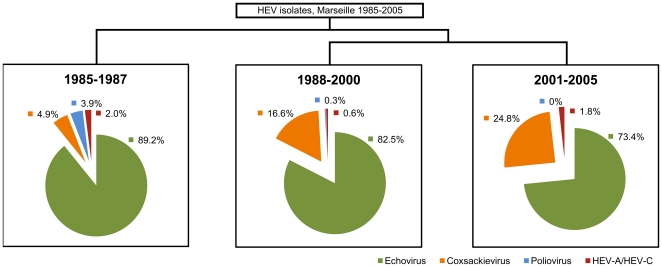
Classification by year of isolation. The Classification and Regression Tree (CART) split the data by year of isolation, describing 3 temporal periods: 1985–1987, 1988–2000 and 2001–2005. The change in proportion was observed for each virus group, with HEV-B further divided into Echoviruses and Coxsackieviruses, while Poliovirus was regarded as separate from HEV-C.

The CART technique classified our data into groups through a series of splits that best differentiated observations of the data (**Figure 3**). The main discriminatory feature was the year of isolation which allowed the definition of 3 temporal periods: 1985–1987, 1988–2000 and 2001–2005. Paying attention to the change in proportion of each virus group across these 3 periods we observed that: (i) Poliovirus has gradually disappeared; (ii) Frequency of HEV-A and other HEV-C and remained consistently low (≤2%); (iii) The proportion of coxsackieviruses has increased from 4.9% to 24.8%, and (iv) The proportion of echoviruses have decreased from 89.2% to 73.5%.

### EV isolation by sample type and cell line

**Figure 4:**
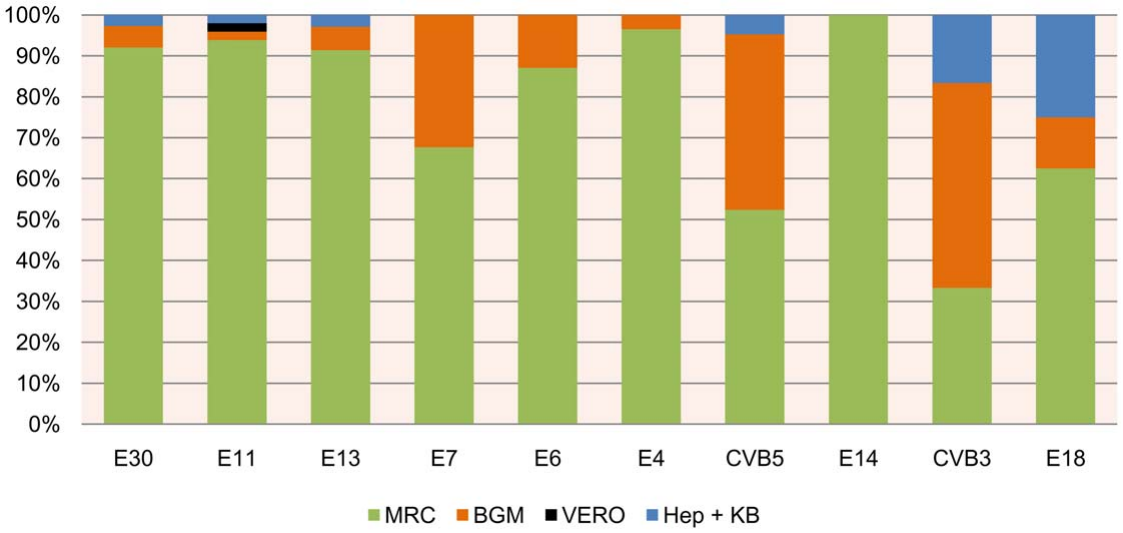
Isolation of the 10 most common Enterovirus serotypes by cell line. MRC5 cells were the most conducive culture line for 84.9% of the samples, with the addition of BGM cell lines enhancing total sample recovery up to 96.3% and covering all serotypes.

Stool is used most frequently in suspected HEV infections over 20 years, save the 2000 and 2005 epidemics during which cerebrospinal fluid (CSF) was highly solicited. Stool samples allowed detection of all serotypes, including most of the non HEV-B serotypes and, notably, all the PV. Further examination of the most common HEV serotypes revealed that 84.9% of the clinical strains showed preferential growth in MRC5 cells, in particular the echoviruses. The additional use of BGM cells enhanced total recovery to 96.3% and allowed better detection of group B coxsackieviruses and Polioviruses (**Figure 4**).

### Accuracy of serotype identification by seroneutralization

The serotypes of 204 clinical samples initially determined at the time of virus isolation by neutralization tests were challenged by VP1 nucleotide sequencing. Only 113 (55.4%) were corroborated by nucleotide sequence. Considering only serotypes with at least 4 strains, this technique was largely accurate (75–91%) for E20, E30, E24, CVB2 and E5. It was average (50–60%) for E14, CVB5, E7, E4 and E6 and poor (26.7–28.6%) for CVA9 and E11. No PV strain was detected using the neutralization technique. The 4 strains that were ultimately designated as PV1 by their VP1 sequences were initially typed as E20, E21, E24 and an adenovirus.

### Phylogenetic analysis of clinical strains

Near full-length VP1 nucleotide sequences (777 nucleotides) of HEV clinical strains were analysed in a phylogenetic tree together with prototype reference sequences and VP1 homologues from NCBI GenBank This overall topology of four distinct clusters corresponding to the four HEV species is consistent with phylogenies previously described [8]. By visualizing the frequency of p-distance scores as a percentage of total scores, clinical VP1 scores fell into three established ranges: variants of the same serotype (≤0.25), sequences of different serotype but of the same species (>0.25 and <0.42) and finally, sequences of different species (≥0.42) (**Figure S1**). Overall, only 0.05%, 0.14% and 0.8% of the three categories respectively were exceptions to these definitions.

### Molecular evolution of EC30 and EC13

**Figure 5:**
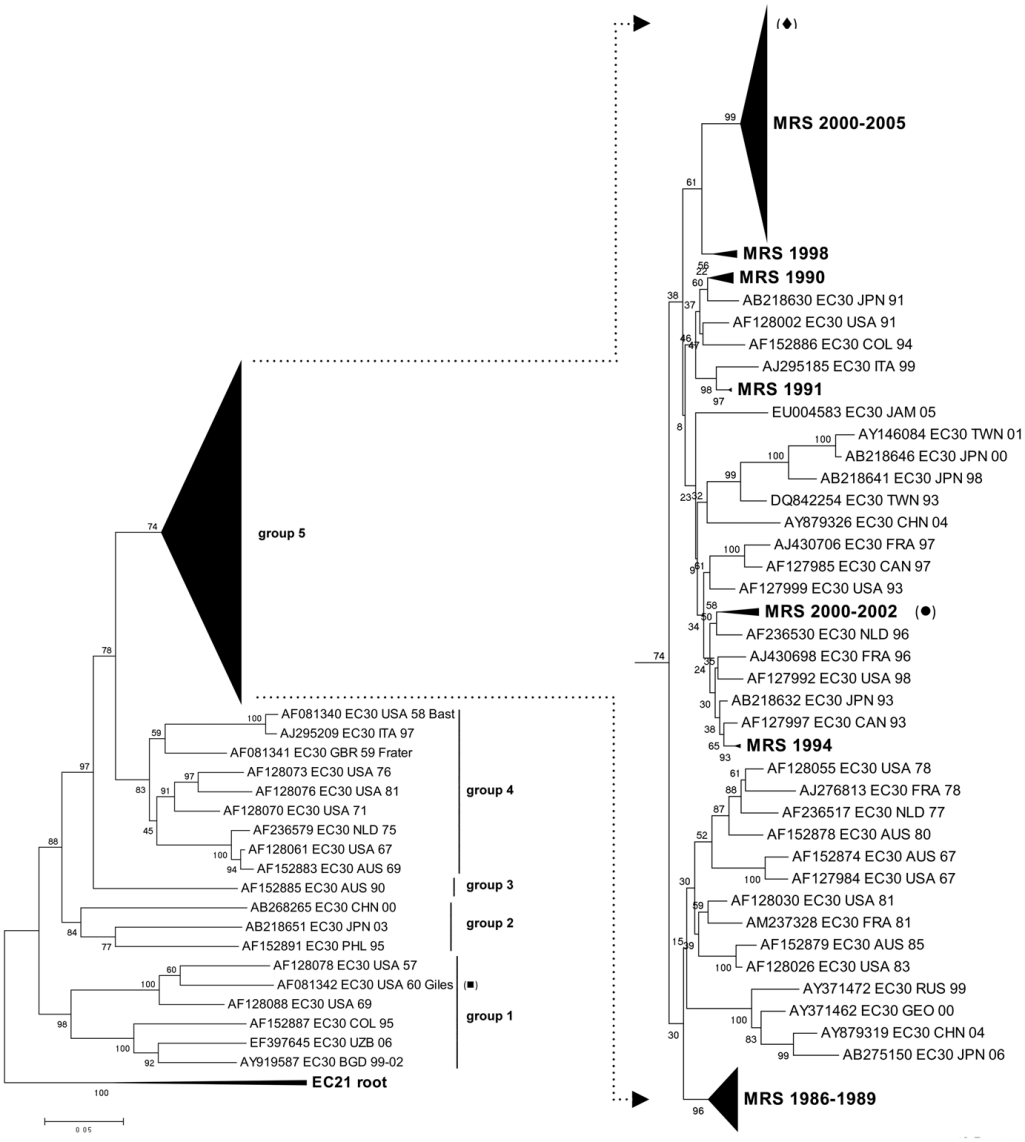
Phylogenetic tree of E30 isolates. 5 groups were observed, with all Marseille isolates clustering in group 5. Closer look at group 5 shows that the most Marseille isolates are genetically related, including all strains from the 2000 and 2005 epidemics. #553 (*diamond*) differs genetically from #497 (*circle*) and prototype Giles (*square*) by 0.157 and 0.312 respectively.

The molecular evolution of E30 was studied in detail by phylogenetic analysis including 159 E30 VP1 sequences from the Marseille database (**Figure 5**). The phylogenetic tree presented 5 temporal clusters, with all Marseille strains clustering together in group 5 (bootstrap 74%) and the majority in a stable subgroup characterized by their period of isolation (2000–2005). Pairwise p-distance showed that the greatest nucleotide disparity between clinical isolates was 0.157, between samples #553 and #497, both isolated in 2002 but which cluster differently in the phylogenetic tree. Notably, the clinical isolate #553 and the prototype strain Giles isolated in 1960 differed genetically by 0.255, which sits just beyond the intra-serotype threshold of 0.25. Its persistent circulation and the extent of its associated epidemics have generated a large genetic diversity within E30, and may go some way to account for this exceptional genetic distance.

**Figure 6:**
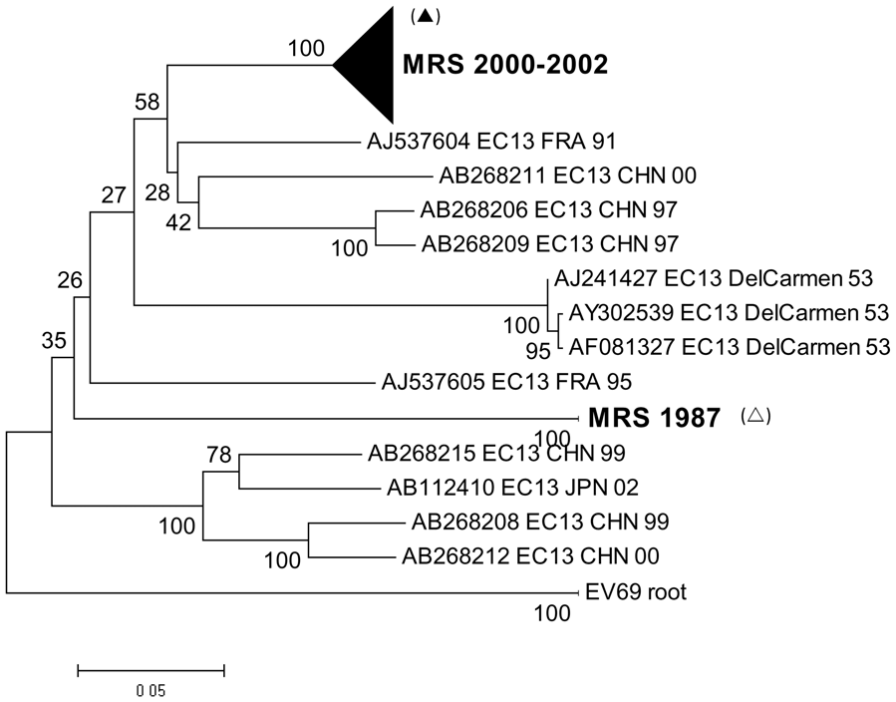
Phylogenetic tree of E13 isolates. All but two sequences were isolated between 2000 and 2002, and clustered in a distinct group. The greatest genetic distance was observed between #369 (*clear triangle*), isolated in 1987, and #375 (*filled triangle*), isolated in 2000.

The molecular evolution of E13 was also further studied by phylogenetic analysis including 36 E13 VP1 sequences from the Marseille database (**Figure 6**). All but two clinical E13 strains were isolated between 2000 and 2002, and clustered in one distinct group together with European and Asian strains from the same period. Within this group, clinical isolates differed in p-distance by no more than 0.036. In contrast, the greatest genetic distance observed between clinical isolates was 0.242, between #369 isolated in 1987, and #375 isolated in 2000.

### Evidence for recombination

**Figure 7:**
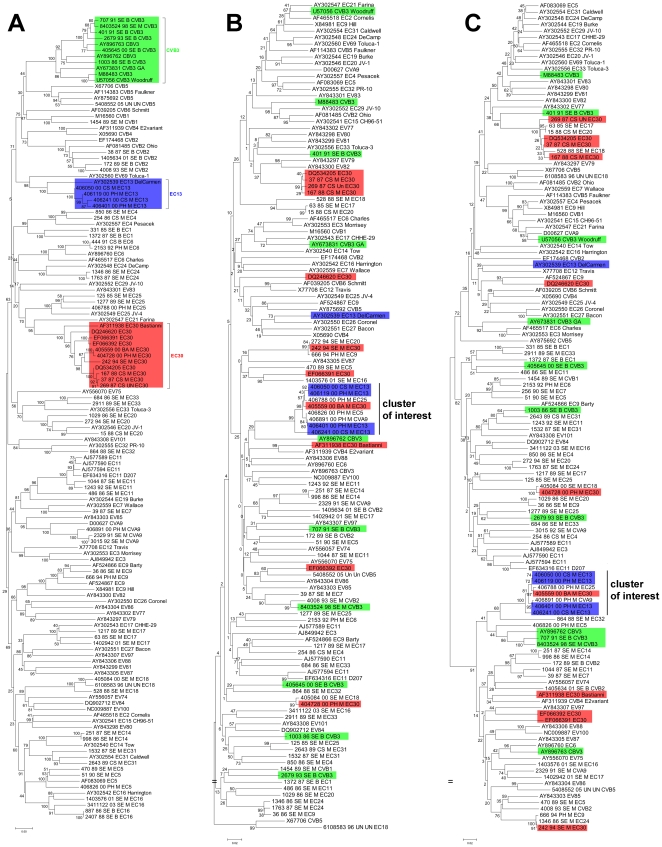
Phylogenetic trees of HEV-B in the VP1 (A), 2C (B) and 3D (C) regions. Inconsistent topologies suggest possible recombination events, especially in the case of E30 (*red*) and CVB3 (*green*). E13 clinical isolates (*blue*) however, differed little genetically across the genome, suggesting the absence of major recombination events. Our cluster of interest includes 7 strains (4 E13, 1 E30, 1 CVA9 and 1 E25) that group reliably (bootstrap 100%) in the 2C and 3D regions but not in the VP1 region.

To examine the extent of intraspecies recombination, we designed primers that targeted portions of the 2C and 3D regions that distinguished HEV-B serotypes phylogenetically from other species. Of 65 HEV-B strains tested, 59 (90.8%) were successfully amplified and sequenced in the 2C region and 61 (93.8%) in the 3D region. Phylogenetic trees were constructed for the VP1, 2C and 3D genes (**Figure 7**). Incongruent tree topologies and inconsistent interserotype clustering show that the genetic relationship between different serotypes is not conserved throughout the genome. The maximum nucleotide distance in the VP1, 2C and 3D regions was 0.42, 0.262 and 0.271 respectively. To reflect this higher level of conservation in the nonstructural region, all p-distance scores were normalized by expressing them as a percentage of the maximal p-distance in nucleotides for each region within HEV-B.

Three serotypes (E13, CVB3 and E30) with more than 4 antigenic variants in our study set were examined to evaluate intraserotype genetic relationships. Regardless of p-distance, all strains clustered according to their serotype in the VP1 region, as is expected since VP1 is the basis of serotype designation. E13 strains differed little genetically in all regions, by 0–8.3% in VP1, 0–14.9% in 2C and 0–7.7% in 3D. Phylogenetic trees also showed consistent grouping in all regions for these strains, all isolated in 2000. There is thus no evidence for recombination among clinical E13 strains in Marseille. In contrast, CVB3 strains were more genetically distant, with 5.2–47.1% in VP1, 52.3–80.2% in 2C and 13.7–81.2% in 3D. Their greater diversity is reinforced by inconsistent clustering across the genome. It is thus highly possible that recombination events have occurred involving the nonstructural region of CVB3 strains. E30 on the other hand, is a more complex case: three strains isolated between 1987 and 1988 demonstrated little divergence with maximum p-distance of 4.3%, 5% and 8.5% in the VP1, 2C and 3D regions respectively and consistent clustering with one another in all regions. However, the remaining E30 strains presented greater genetic distance, by 0–20.7% in VP1, 64.1–75.2% in 2C and 62–88.6% in 3D. This disparity is also observed in their variable phylogenetic positions across the genome.

This pattern is exemplified by E30 strains #405559 and #404728, both isolated in 2000 and which are identical (p-distance  = 0%) in their VP1 nucleotide sequence but differ by 73.3% and 74.2% in their 2C and 3D sequences respectively. Interestingly, the exact opposite clustering pattern was observed in 7 strains: 1 E30 (#406559), 1 CVA9 (#406891), 1 E25 (#406788) and 4 E13 (#406401, #406050, #406119 and #406241) strains differed by a maximum 92.6% in their VP1 region, but only by 14.9% in 2C and 8.5% in 3D. Phylogenetic analysis showed reliable grouping (bootstrap  = 100%) in both the 2C and 3D regions for our cluster of interest.

## Discussion

The present study describes the frequency of enteroviral serotypes isolated in Marseille between 1985 and 2005. Enteroviruses are known to circulate in the summer and autumn months in temperate regions [19], [29]–[30]. In Marseille, we observed that HEV isolation peaked in the spring and summer months, notably during the 2000 and 2005 epidemics when unusual levels of HEV activity were detected as early as May. The last natural case of poliovirus in Marseille was described in 1988, in line with its complete elimination in France and the European continent in 1990 [31]. E30 was the most frequent enterovirus isolated in Marseille, in accordance with epidemiological data collected by the RSE, the sentinel laboratory network for the surveillance of Enteroviruses in France [30]. This high incidence also reflects similar circulation levels and the occurrence of E30-associated aseptic meningitis epidemics in 2000–2001 in North and South America, Europe and Asia [23]–[24], [32]. The general distribution of E30 is temporally consistent with other European strains included in our analysis, and supports a microevolution as a continuous cline with rare re-emergence of more ancient strains. Unlike E30, E13 was considered a rare serotype with no outbreaks associated with this virus and had only ever been isolated once in Marseille prior to 2000. Its sudden emergence as a predominant serotype was also observed in other countries: in the United States, E13 accounted for 24% of all reported HEV isolates in 2001, compared to 1.6% in 2000 [33]. In Japan, E13 had only been isolated once before 2001, during which 65 strains were isolated [34]. E13 was first identified in Spain during an aseptic meningitis outbreak in 2000 [35].

Regarding the strategies and methodologies used during the study period for detection and characterization of enteroviruses, a number of observations could be made. Firstly, stool has been the most frequently used sample type for enterovirus isolation, and the most useful since it allowed the isolation of all serotypes. Secondly, MRC5 was the most conducive cell line for enterovirus isolation, and coupling with BGM cell line, resulted in a more extensive coverage for HEV-B strains. Thirdly, seroneutralization-based HEV typing showed 55.4% accuracy when compared to VP1 sequence analysis. This divergence in identification may be explained in part by technical insufficiency of the seroneutralization typing protocol (*e.g.* cross reacting activity, use of pools raised against strains prevalent more than 30 years ago). Another possible explanation could be the presence of more than 1 serotype in a patient sample, whereby the dominant serotype during its reproduction in culture for VP1 sequencing is different from the dominant serotype during initial culture for diagnostic seroneutralization. It has also been highlighted that poliovirus might be present in working stocks of other viruses, even when unambiguously identified and labeled [36].

The validity of VP1 serotyping protocol and pairwise genetic distance analysis has been primarily established with enterovirus prototype sequences or with clinical sequences spanning a short period of time [7]. Our analysis of a large number of clinical isolates over 20 years reinforces the pertinence of this technique that allows the identification of most HEV sequences using the simple computationally non-intensive genetic distance calculation. Furthermore, in the few instances whereby the genetic diversity within a serotype can be so significant as to exceed the 0.25 threshold, as observed in E30, genetic distance can be coupled with the phylogenetic analysis of VP1 to provide a non-ambiguous identification of HEV, a strategy previously validated with the delineation of Hepatitis C virus genotypes [37]–[38].

The serotypic identification of enteroviruses is challenged by the existence of recombination events [39]–[40]. Isolates sharing similar VP1 genes but differing in other parts of the genome may display different epidemiological or clinical properties. Phylogenetic topologies of different portions of the enteroviral genome describe HEV strains with genetically consistent VP1 regions and more interchangeable 2C and 3D regions, particularly demonstrated by E30 and CVB3 strains from Marseille. This suggests that closely related VP1 regions can be associated with divergent 2C and 3D regions. The mechanism of RNA recombination in enteroviruses is commonly accepted to involve template switching during RNA synthesis, with recombination points most frequently identified within the nonstructural region [28], [41]. In contrast, we observed the opposite trend in an unusual cluster of 7 strains: #406891_CVA9, #406401_E13, #406050_E13, #406119_E13, #406241_E13, #406788_E25 and #406559_E30 showed little similarity in the VP1 region, but a marked resemblance in the nonstructural region. This suggests the circulation of highly similar HEV strains which differ primarily in the region by which they are attributed serotypes. Considering that all 7 strains were isolated during the 2000 Marseille epidemic during which the E30 and E13 serotypes were particularly prominent, this genetic similarity could explain the emergence of E13 as an epidemic serotype by a recombination between circulating E13 and epidemic E30 strains. This might also account for the lack of direct correlation between serotype and pathology, such as how several (VP1-defined) serotypes can provoke the same clinical manifestations. A new model of enteroviral genetics has been suggested, such that enteroviruses should be regarded as a pool of independently evolving genomic fragments [42]. We show that clinical strains of enteroviruses circulating over 20 years lend credence to this model by showing the inadequacies of the current model of demarcated serotypes.

In this work, serotypes from the HEV-B species account for 98% of all isolates in Marseille. This echoes HEV-B levels described in Spain (89.3%) [43], in the United States (89.5%) [19] and in Tunisia (92.2%) [44], studies which also used cell culture to first isolate the virus in the typing process. However, the use of cell culture techniques may distort any derived epidemiological data since some HEV serotypes (Coxsackievirus A and certain numbered HEVs) do not grow or grow poorly in cell culture, and suggests that the proportion of circulating non HEV-B serotypes has been underestimated. Such a study of clinical samples in a clinical virology laboratory is sure to encounter some limitations, in part by the bias associated with cell culture techniques, but also by other factors such as patient sample referral and enteroviral disease presentations that might not be actively investigated. This work should thus be more accurately regarded as a clinical profile of HEV-B serotypes in Marseille. As such, we feel the need to reinforce efforts for identifying HEV directly from clinical samples, bypassing the need for cell culture systems.

## Materials and Methods

### Marseille HEV strain collection and sequence database

#### Enterovirus samples

All samples taken for diagnostic purposes are accessible for research under French national regulations regarding biomedical research (Loi Huriet-Sérusclat (loi 881138)) without requiring neither specific written consent from the patient nor approval from an ethics committee. All clinical samples were obtained from the Laboratory of Virology, University Hospital La Timone (Marseille, France) from 1985 to 2005. Specimen types comprised of nasopharyngeal swabs, stool, cerebrospinal fluid, saliva and bronchoarterial specimens.

#### Cell lines

MRC5 cells (Human fetal lung fibroblasts) were cultured in Basal Medium Eagle (BME), 10% decomplemented Fetal Bovine Serum (dFBS), 1% L-Glutamine (L-GLN), 1% Penicillin-Streptomycin (PS). Hep2 (Human laryngeal carcinoma cells), KB (Human laryngeal carcinoma cells), Vero (African green monkey kidney cells) and BGM (Buffalo green monkey kidney cells) cell lines were grown in Minimum Essential Medium Eagle (MEM), 5% dFBS, 1% L-GLN, 1% PS. All cell cultures were incubated at 37°C under 5% CO_2_.

#### Enterovirus diagnosis

(i) Prior to 2000, HEV diagnosis consisted of growing samples in cell culture in MRC5, BGM, Vero and KB cell lines. Once cytopathic effect (CPE) was observed, the presence of HEV antigen was tested by immunofluorescence with a monoclonal mouse anti-EV antibody (clone 5-D8/1, Dako) and a secondary goat anti-IgG mouse fluorescein conjugate [45]; (ii) From 2000 to 2004, diagnosis was achieved by classic RT-PCR using the Enterovirus Consensus Kit 5 (Argene) and inoculation of samples onto MRC5, BGM, Vero and Hep2 cell lines which were similarly evaluated by CPE and immunofluorescence; (iii) From 2005 onwards, a real-time pan-enterovirus RT-PCR was used (adapted from [5]) along with the inoculation of samples onto MRC5, BGM, Vero and Hep2 cell lines which were similarly evaluated by CPE and immunofluorescence. For all samples, the cell line in which CPE was most rapidly observed was recorded and the virus isolates stored in the Marseille Public Hospitals virus collection. Globally, from 1985 to 2005, the same cell culture detection and isolation protocol was performed based on the use of MRC5, Vero and BGM cells, which represent 96.5% of all isolates (cf Results section). The only change during the period was the replacement of KB with Hep2 cells from 2000 onwards, and which represent 0.5% and 3% of all isolates respectively).

#### Seroneutralization

A portion of HEV-positive samples (n = 204) processed between 1985 and 1994 were typed by seroneutralization with Lim-Benyesh-Melnick antiserum pools [6].

#### VP1 Sequencing

Strains recorded in the Marseille HEV collection were reproduced in the cell line in which they were originally isolated from culture. Supernatant was clarified by centrifugation and extracted using the EZ1 Virus Mini Kit (Virus Card 2.0) in an EZ1 BioRobot (QIAGEN) according to the manufacturer’s protocol. Reverse transcription was carried out with Reverse Transcriptase MultiScribe (Applied Biosystems) with random hexamers. Each viral cDNA was then amplified by nested *Taq* DNA Polymerase PCR (Invitrogen) using 2 VP1-specific primer pairs (adapted from [7]). Amplification products were visualized by 2% agarose gel electrophoresis and ethidium bromide staining, then purified with QIAquick PCR Extraction or Gel Extraction kits (QIAGEN). Sequencing was carried out using a Big Dye Terminator Cycle Sequencing Reaction kit and an ABI Prism 3130 DNA Sequencer (Applied Biosystems).

#### Marseille HEV sequence database

VP1 sequences were obtained and their serotype identified by phylogenetic analysis (n = 654). They were archived in the Marseille HEV VP1 database in the following format: #Reference number_Year_Sample type_Cell line_Serotype. No written patient consent was required as all strains were characterized for etiological purposes.

#### 2C and 3D sequencing

HEV-B strains from the HEV database (n = 65) were chosen to be representative of serotypes and years for the period surveyed. 2C, coding region for the helicase/NTPase; and 3D, coding region for the RNA-dependent RNA polymerase, were chosen as representative of the P2 and P3 regions respectively. We designed primer pairs to specifically amplify HEV-B serotypes, targeting portion of the 2C and 3D regions that phylogenetically distinguished HEV-B from other HEV species. RT-PCR was performed as described above, using the specific primer pairs 2C-F (TTYGAYGGiTAYAARCARCA) and 2C-R (GGiCCYTGRAAiARiGCYTC) or 3D-2F (TTYTGGWSiAARATHCCiGT) and 3D-R (CKiACRTGRTCYTGiGTRTT). Amplification products were visualized, purified and sequenced as described above.

### Sequence analysis

#### Phylogenetic analysis

All sequence chromatograms were analysed with Sequencher 4 software (Gene Codes Corporation). Multiple sequence alignments were realized with EBI ClustalW2 (http://www.ebi.ac.uk/Tools/clustalw2/index.html) [46] on default settings and manually edited with BioEdit [47]. The nucleotide sequences were translated into and aligned as amino acids. Using the programme DAMBE (http://dambe.bio.uottawa.ca/dambe.asp) [48], nucleotide sequences were aligned against the amino acid sequences. Phylogenetic trees were constructed with MEGA version 3.1 (Molecular Evolutionary Genetics Analysis) [49]. For VP1, 2C and 3D, this was achieved using the Neighbor-Joining method on a Jukes-Cantor model. Partial VP1 sequences (<400 nucleotides) were omitted from phylogenetic analysis. Pairwise distance matrices were drawn to calculate p-distance, the proportion of nucleotide sites at which the two sequences differ for the totality of the sites compared. The consistency of tree topologies was tested by bootstrapping in 1000 pseudoreplicates.

### Statistical analysis of epidemiological data

A Classification and Regression Tree (CART) was established to determine characteristic features of the dataset as a series of if-then logical conditions [50]. The monthly frequency of Enterovirus isolations was plotted on a control chart for count data (Poisson distribution) estimating an upper control limit with µ ±3σ (99.73% confidence) [51]–[52]. Statistical analysis was carried out using the R.2.10.1 environment (http://www.r-project.org) and the qcc package [53].

## Supporting Information

Figure S1 **Pairwise p-distance scores of clinical Enterovirus VP1 sequences, 1985-2005.** 20 years of clinical strains validated the three established ranges of genetic distance that indicate variants of the same serotype (≤0.25), sequences of different serotypes but the same species (>0.25 and <0.42), or sequences of different species (≥0.42).
(TIF)
