# Supplementary figures and images for: A Retrospective Overview of Enterovirus Infection Diagnosis and Molecular Epidemiology in the Public Hospitals of Marseille, France (1985–2005)
Source: PLoS One. 2011 Mar 18;6(3):e18022. doi: 10.1371/journal.pone.0018022 (PMC3060927; doi:10.1371/journal.pone.0018022)

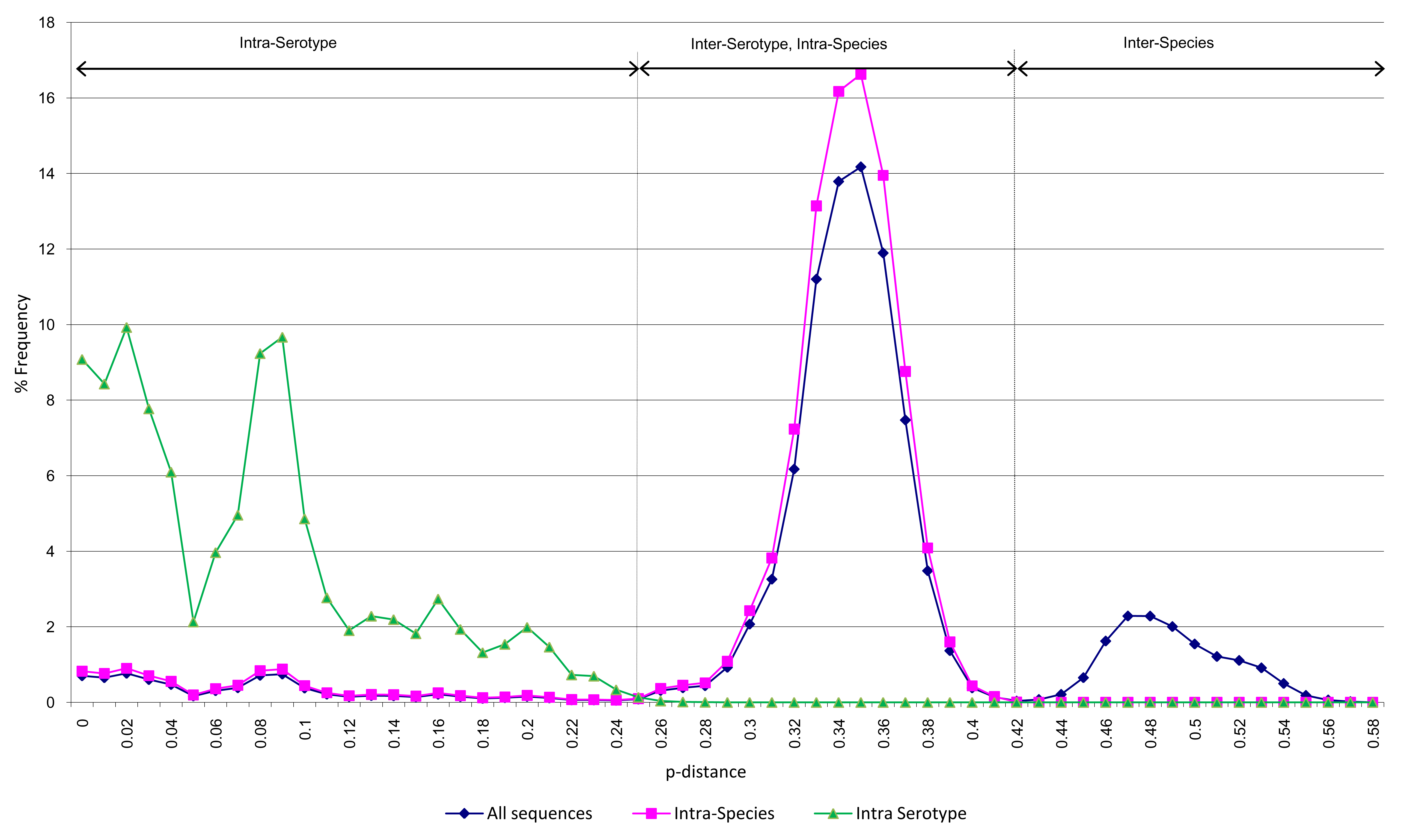

Supplement: Figure S1 — Pairwise p-distance scores of clinical Enterovirus VP1 sequences, 1985-2005. 20 years of clinical strains validated the three established ranges of genetic distance that indicate variants of the same serotype (≤0.25), sequences of different serotypes but the same species (>0.25 and <0.42), or sequences of different species (≥0.42). (TIF) [file pone.0018022.s001.tif]
